# Automated quantification of myocardial perfusion based on segmentation and non-rigid registration of contrast-enhanced cardiac magnetic resonance images

**DOI:** 10.1186/1532-429X-13-S1-P91

**Published:** 2011-02-02

**Authors:** Giacomo Tarroni, Cristiana Corsi, Federico Veronesi, James Walter, Claudio Lamberti, Roberto M Lang, Victor Mor-Avi, Amit R Patel

**Affiliations:** 1 University of Bologna, Bologna, Italy; 2 University of Chicago, Chicago, IL, USA

## Introduction

Quantification of myocardial perfusion from cardiac magnetic resonance (CMR) images relies on manual tracing of myocardial regions of interest (ROIs) and their repositioning frame-by-frame throughout the contrast-enhanced image sequence. Additionally, out-of-plane motion due to patient’s respiration frequently requires that myocardial ROIs to be redrawn.This tedious and potentially inaccurate methodology hinders widespread clinical use of imaging-based quantification of myocardial perfusion.

## Purpose

We developed a technique for automated identification and registration of myocardial ROIs from CMR images as a basis for perfusion quantification.

## Methods

Our approach uses statistical and edge-based level-set methods for endocardial and epicardial border detection, which is performed in an automatically chosen single frame. To take into account the changes in position, size and shape of the myocardium, we developed an algorithm for non-rigid registration using a 2D multi-scale extension of normalized cross-correlation (figure, left). This approach was tested on 66 ECG-gated short-axis image sequences obtained in 11 patients at rest and during regadenoson stress at basal, mid and apical levels during first pass of a Gadolinium-DTPA bolus. (Philips 1.5T scanner with a hybrid gradient echo/echo planar imaging sequence: nonselective 90° saturation pulse followed by 80 ms delay, voxel size ~2.5x2.5mm, slice thickness 10mm). Standard myocardial ROIs were automatically defined and registered throughout the image sequence. Pixel intensity was measured in each segment over time, resulting in segmental contrast enhancement curves.

## Results

**Figure 1:**
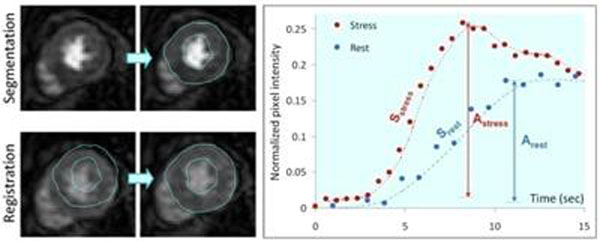
Example of automated detection of the myocardium (top left), followed by non-rigid registration (bottom left). Time curves automatically derived from the first-pass image sequence (right) at rest and regadenoson stress depict the expected differences in peak-to-peak amplitude (A) and contrast inflow rate (S).

Analysis of one sequence required <1 min and resulted in endo- and epicardial boundaries that were judged accurate in all image sequences. Time-curves showed the typical pattern of first-pass perfusion with signal-to-noise ratios of 15±5 at rest and 19±4 during stress. During stress, contrast inflow rate (0.031±0.013 vs 0.014±0.004 sec^-1^, p<0.05) and peak-to-peak amplitude (0.20±0.05 vs 0.14±0.03, p<0.05) were both increased compared to rest (figure 1, right).

## Conclusions

Despite the extreme dynamic nature of contrast enhanced image sequences and respiratory motion, fast automated detection of myocardial segments and quantification of tissue contrast results in curves with excellent noise levels, which reflect the expected effects of stress. The application of this technique could thus address the strong clinical need for automatic detection and quantitative evaluation of myocardial perfusion.

